# A Case of Mollities Ossium during the Growth of the Body

**Published:** 1805-05-01

**Authors:** 

**Affiliations:** Newcastle


### A Case of Mollities Ossium during the Growth of the Body; *communicated by* Dr. Trotter, *of New-castle.*

W. T. a healthy, active boy, in his fifteenth year, began to grow exceedingly fast; so that in the space of twelve months, his height was increased from sixteen to eighteen inches. With this he looked slender, was extremely thin, and his head stooped, and rather inclined to the one shoulder. He now felt himself very weak, and was unequal to any kind of exercise, from the sudden fatigue which it induc d. During all this change of appearance, no medical person was consulted, and his father and mother, of their own accord, thought sea-bathing would be the means of restoring his strength, as the rapid growth of the body seemed the sole cause of the debility, without any sign of disease. The

The sea-bathing, at first, was thought to have considerable effect, and he appeared to mend, when, all at once, his appetite failed, the debility increased, and he was incapable to sit up. It was now that pain and uneasiness were felt about the second *cervical vertebra*; and he was unable to sustain his head. In this condition he was brought home; his height upwards of six feet. I saw him on the 24th of August last: at that time he laboured under an almost *general paralysis* of all the muscles of the body, except those of the eyes, nose, and ear; even the muscles of the tongue were affected, and deglutition was difficult. It was plain that the muscular power was impaired, so far as it depended on free communication with the nerves originating from the *medulla spinalis*. The desire for food was almost gone; the intestinal canal was so torpid, that nothing but active purgatives kept the body open; and the urine was voided with the utmost difficulty, in small quantity. The *dyspnœa* which attended was peculiar; not like that of the phthisical or asthmatic state, or what is observed in pneumonic inflammation; but was free or laborious at unequal intervals. The pulse was singular in its motions, alternately quick and slow, distinct or irregular, with, sometimes, long intermissions. He lay in the horizontal position; every limb was immovable, and could not turn the head a single inch to either side.

From the wry head, and the general immobility of the muscles, I was naturally led to examine what was called a swelling in the neck. The thinness of the flesh made the diseased part appear very plain. The tumor was certainly osseous; the head, with the *atlas*, was thrown forward, and the *processus dentatus* of the second *vertebra* could be distinctly felt, so that a partial dislocation of these bones had taken place. Independent of compression on the spinal marrow, from this cause it was probable, there was also some deposition of bony matter, or elongation of the intervertebral cartilage; for it was not known how long these bones had been affected. On the whole, from the symptoms enumerated, I was inclined to form a very unfavourable *prognosis*; and I could not help informing the parents of the youth, that the danger was imminent.

The treatment which I recommended was chiefly directed to *strengthen the body*. The diet was ordered to be as nutritious as the deficient appetite would allow, and to consist of animal matter. A *smart blister* was immediately laid over the *vertebra dentata*, and stimulating applications were were rubbed along the spine. A mixture of *decoct. cinchonæ, sp. æther. nitros. sp. ammon. comp.* and *cret. præparat.* with a draught of *tinct. opii* at bed-time, were the medicines. The *carbonate of lime* was partly suggested to correct acidity; but it might be beneficial in supplying calcareous substance to the imperfectly hardened bones. Some experiments of naturalists inform us, that fowls lay eggs without shells, if they are deprived of gravel, or food that does not yield calcareous matter; so, in the rapid growth of a bone, a similar deficiency may be suspected.

Under this treatment, the powers of Nature so far improved, that in ten days the danger of life disappeared; the sleep became refreshing, the appetite increased, the bowels regular, urine made with ease, and the pulse distinct and equal. On the right side, the power of motion so far returned, that he could raise the arm to his mouth; but, from that time, little further recovery has taken place; and there is every reason to believe, that the spinal marrow is permanently compressed; at least, his parents seem to look for no farther medical aid.

I have called this case *mollities ossium*, without any reference to a diseased state of the bones: I only mean, that they are soft, from rapid growth. The *cervical vertebræ* were evidently incapable of supporting the weight of the head; and from the office of the *processus dentatus*, being chiefly for that purpose, by its soft consistence, joined to the weakened ligaments and muscles, the *atlas* was partially thrown from its situation. This is not a common case; Nature seldom leaves her work so imperfect. I apprehend something might have been done, in the early stage of the complaint, by mechanical means: some sort of prop, or lever, to sustain the head equally between the shoulders, so as to ease the *cervical vertebræ*, I think, might be still serviceable. I should be glad to have the opinion of your Surgical Correspondents on the subject.

The patient is a most promising young man: has made considerable progress in Grecian literature, and excels in the knowledge of the Latin and French languages.

Here is a fine illustration of that sympathy of parts, which is spread by means of nervous communication: the loss of motion, in the muscles which turn the head; difficult deglutition; unusual dyspnœa; impeded motion of the heart; anorexia; constipation; dysuria, &c. all of which may be referred to those frequent inosculations which the eighth, ninth, and tenth pairs of nerves of the head head have with those of the cervical and spinal nerves, forming a vast circle of smpathetic feelings.

*April 4th*, 1805.

P. S. I am much obliged to your ingenious correspondent, Mr. Fogo, for his explanation of my case of the lady, in 68. By turning to it, he will find, I did not mean to defend the designation. It was only proper, in publishing it, to give it a name. A man of Mr. Fogo's systematic ideas of pathology, could never have committed such a blunder as to call it *pleurisy*.

